# Effects of discharge education program on recovery and quality of life after cardiac surgery: a 12 week follow-up single-blind randomized controlled trial

**DOI:** 10.1186/s12893-026-03939-4

**Published:** 2026-06-16

**Authors:** Eva Kajti, İsmail Selçuk, Ümmü Nehir Selçuk, Hamdi Toköz, Ayfer Özbaş

**Affiliations:** 1 Istanbul Atlas University, Istanbul, Türkiye; 2 Istanbul Dr. Siyami Ersek Chest and Cardiovascular Surgery Training and Research Hospital, Istanbul, Türkiye; 3 Atasehir Acibadem Private Hospital, Istanbul, Türkiye; 4 Demiroglu Bilim University Florence Nightingale School of Nursing, Istanbul, Türkiye

**Keywords:** Cardiovascular diseases, quality of life, nursing care

## Abstract

**Aims:**

In the contemporary era of rapid societal and technological advancement, factors such as sedentary lifestyles, environmental pollution, unhealthy dietary habits, consumption of sugar-sweetened beverages, tobacco and alcohol use, and the growing prevalence of comorbid conditions associated with these risk factors have contributed to cardiovascular diseases becoming one of the leading causes of mortality worldwide. Among the therapeutic interventions for cardiovascular diseases, open-heart surgery remains one of the principal treatment modalities, as it is associated with superior symptom control, enhanced quality of life, and improved long-term survival outcomes compared with medical management alone. Beside the surgical intervention one of the main components determining the outcomes of the surgery is the patient education therefore this study was conducted to determine the effect of discharge education provided after cardiac surgery on recovery and quality of life.

**Methods and results:**

This research was conducted as a randomized pretest-posttest controlled study to determine the effects of discharge education after cardiac surgery on recovery and quality of life. The study was conducted between March 2022 and July 2023, in two Training and Research Hospitals with 70 inpatients. The data of the study were collected face to face and via telephone calls using the ‘Patient’s Personal Information Form’, ‘Multidimensional Index of Life Quality-MILQ-TR’, ‘Quality of Recovery − 40 Questionnaire-QOR-40’ and ‘Telephone Follow-Up Form’. According to this study’s results, the recovery quality level and the quality of life of the patients in the intervention group was significantly higher than the patients in the control group.

**Conclusion:**

According to the results of the study, which was evaluated at a 95% confidence interval and a significance level below *p* < 0.05; the quality of recovery and quality of life of the intervention group patients were statistically higher, the complications were fewer, and the level of knowledge was higher.

**Trial registration:**

Trial Registry The Effects of Discharge Education Program on Recovery and Quality of Life After Cardiac Surgery. Unique Identifying Number NCT05631340. Date of Registration 21/11/2022. ( https://clinicaltrials.gov/study/NCT05631340 )

**Supplementary Information:**

The online version contains supplementary material available at 10.1186/s12893-026-03939-4.

## Introduction

In nowadays rapidly developing world, sedentary lifestyle, air pollution, fast food, sugary beverages, tobacco and alcohol use, and the increase in comorbidities related to these risk factors have led cardiovascular diseases (CVD) to be a primary cause of mortality [1, 2]. According to World Health Organization, 113 million people were living with CVD in 2019 and 12.7 million new diagnoses were added. When we look at the situation in Turkey, the incidence of CVD was 0.6% in 2017, meaning that 663 people out of every 100,000 citizens were diagnosed with CVD [1]. The latest data show that, even in 2022, CVDs ranked first among the most common non-transmittable diseases with 17.9 million deaths annually [3]. According to the American Heart Association, there are seven modifiable factors that increase the risk of coronary artery disease (CAD). These factors are defined as the " Life’s Simple 7” and they are; smoking, insufficient physical activity, unhealthy diet, obesity, hyperlipidaemia, diabetes and high blood pressure [4]. On the other hand, factors such as; male gender, race/ethnicity, age and genetic status are unchangeable risk factors of CAD [4].

Nowadays preventive health services related to CVD are at the forefront, but when the disease develops, treatment approaches are addressed in three groups. The first of these is reducing risk factors, secondly medical treatment and thirdly surgical treatment. Of these approaches, surgical treatment is preferred because it provides a symptom-free, high-quality and longer life expectancy compared to medical treatment [5, 6]. Surgical methods applied in cardiovascular diseases can be listed as coronary artery bypass grafting (CABG), valve repair, valve replacement, removal of tumors in the heart and heart transplantation [7]. Patient education given postoperatively is defined as providing information on health behaviours affected by beliefs, values and motivation or obtaining, assimilating and transforming health-related information into behaviours [8]. Patient education is an important tool for increasing compliance and satisfaction, reducing costs, reducing morbidity and mortality, improving quality of life and increasing patients’ independency levels [9]. “Quality of Life-QOL” concept in healthcare, has attracted attention especially with the acceleration of cancer treatments, and the success or failure of a clinical treatment, in addition to biological and demographic indicators, has begun to be evaluated with the positive or negative statements that patients attribute to their QOL and quality of recovery. These subjective evaluations made by patients are gathered under a single roof named “quality of life” [10].

Although discharge education in cardiac surgery is of critical importance it is noticed that even in the main hospitals in Türkiye there is a lack of written or audio and video materials. To address this gap a booklet and video materials including a mobile application was developed and presented to the patients to profit from them in the postoperative process.

## Materials and methods

This study was conducted in the inpatient clinics of the Cardiovascular Surgery Departments of two Training and Research Hospitals between March 2022 and July 2023.

Inpatient clinics in hospitals provide 24-hour service and generally work in shifts between 08:00–16:30 during the day and 16:30 − 08:00 at night. During the study, 3 nurses and 1 caregiver were working during the day, and 2 nurses and 1 caregiver were working during the night shift in both hospitals. Interviews with patients and families were conducted during weekend since most of the discharges in both hospitals were done at the beginning of the week. Postoperative patients on the 5th-7th day after surgery, who met the inclusion criteria, and agreed to participate in the study were interviewed. The “Patient’s Personal Information Form” developed in line with the literature by the researcher (11; 1), MILQ-TR (Cronbach’s alpha = 0.94), QoR-40 (Cronbach’s alpha = 0,91) and the “Telephone Follow-up Form” were used as data collection tools. The researcher formulated three hypotheses regarding the data and results of the patients gathered after 12 weeks follow up:

- H1_1_: Patients who receive discharge education following cardiac surgery experience faster recovery compared with those who do not receive discharge education.
- H1_2_: Patients who receive discharge education following cardiac surgery experience fewer complications compared with those who do not receive discharge education.
- H1_3_: Patients who receive discharge education following cardiac surgery demonstrate higher quality of life compared with those who do not receive discharge education.

All data and results in this clinical trial were reported accordingly with CONSORT Guidelines.

### Sampling

Since no similar study was found in the literature, a power analysis was performed by using G*Power (v3.1.7) program to determine the sample size. Based on the mean scores of the Multidimensional Quality of Life Scale (MILQ-TR), the reliability and validity of which was studied by Demir and Özer (2021), it was decided to take 35 people for each group and to conduct the research with a total of 70 people. The randomization of the study planned as a single-blind (patients), randomized controlled study was done using the “Permuted Block Randomization’’. Six subject (patient) blocks were stratified in 10 blocks according to the permutation method. Randomization started from the tenth block and can be used again starting from the tenth block for a sample of over 60 patients.

### The application process of the study

This study, which was planned to be conducted in two separate intervention and control groups, was explained to the patients who met the inclusion criteria. After verbal and written consent eligible patients (being 18 years of age or older; able to read, write, understand, and communicate in Turkish; having no visual, hearing, or cognitive impairments; being conscious, oriented, cooperative, and open to communication) who voluntarily agreed to participate were enrolled and data from the patients were collected by using the above-mentioned data collection tools. Patients undergoing cardiac surgeries that did not include a thoracotomy were excluded.

#### Ethical issues

The study was approved by the local Clinical Research Ethics Committee of a Training and Research Hospital (Decision date and number, 04. 10.2021/HNEAH-KAEK 2021/236). Written permission was granted by both institutions where the study is performed. Informed written consents to participate were given voluntarily by the patients and their caregiver. All procedures in this study are conducted in accordance with the human ethics standards of the institutional and national research committee and with the 1964 Helsinki declaration and its later amendments or comparable ethical standards. The study has a Clinical Trials record (NCT05631340) dated 21/11/2022 (https://clinicaltrials.gov/study/NCT05631340)*.*

### Applications performed on intervention group

Firstly the ‘Patient’s Personal Information Form’ was filled out by interviewing the patient. Information such as, patient’s height and weight, ASA, type of surgery, age, extubating time, discharge time from the CVICU, etc. was obtained from the patient’s medical file. It took 5–10 min to fill out the form. The patients were asked to fill out the MILQ TR and the QoR-40 after being explained to them. Finally, the patients were asked to answer the questions on the ‘Discharge Education Knowledge Level Form-(DTKLF)’. The patient’s discharge education was explained with the help of a digital presentation and the booklet, the necessary parts were repeated, and the patient was asked questions to ensure that they understood the education. The discharge education prepared based on Daily Life Activities included topics as: safe environment, communication with the hospital staff, sleeping, breathing, feeding, toileting, working, sexuality and end of life.

The explanation of the education took approximately 20–25 min. After the explanation, the second DTKLF was filled out, the education booklet and mobile application prepared by the researcher and approved by 7 reviewers, were presented to the patient and the interview was concluded. The patients were followed up by the researcher by calling them on the 10th day, 4th, 8th and 12th weeks after discharge. During the phone call, information such as the course of vital signs, complications, hospital submissions or re-hospitalizations were questioned and recorded on the relevant form. The discharge education was repeated accordingly to the patient’s needs and/or the complications they experienced. In the last interview (12th week), the MILQ-TR, QOR-40 and the DTKLF were reapplied.

### Applications performed on control group

After fulfilling Patient’s Personal Information Form, MILQ TR and QoR-40 the patients were asked to answer the questions on the DTKLF. Without giving any additional education to that of the hospital, the patients were informed that they would be called a total of 4 times, on the 10th day, 4th, 8th and 12th weeks after discharge. In the last interview the MILQ-TR, QOR-40 and the DTKLF were reapplied.

### Data analysis and evaluation

To evaluate the findings and results of the study, SPSS (Statistical Package for the Social Sciences) version 26.0 (IBM Corp., Armonk, NY, USA) program was used for advanced statistical analysis. The reliability level of the measurement items was determined with Cronbach alpha coefficient. Categorical variables were presented as frequency (n, %) and continuous variables were presented as mean ± standard deviation, median value and range values (minimum-maximum). Independent samples t-test was used for comparisons between two groups for quantitative variables with normal distribution, while the Mann–Whitney U test was used for quantitative variables that did not show normal distribution. For within-group comparisons of three or more repeated measurements of variables that were not normally distributed, the Friedman test was applied, and pairwise comparisons were evaluated using the Wilcoxon signed-ranks test. Chi-square tests (Pearson chi-square test, Yates’ continuity correction chi-square test, and Fisher’s exact chi-square test) were used for the comparison of qualitative data. The results were evaluated at a 95% confidence interval and significance level *p* < 0.05.

## Results

The mean age of the patients was 61.20 ± 9.06 years, 77.1% were male, 88.6% were married, 70.0% had high school or lower degree, 37.1% were currently employed, 85.7% lived in the district, 95.7% had a medium income, 51.4% lived with their spouses and children, 82.9% had a diagnosed additional disease, 81.4% used a regular medication, 70.0% smoked, 18.6% used alcohol, 67.1% had a family history of open heart surgery, and 77.1% had previous hospitalization experience. CABG surgery was performed on 87.1% of the patients, and 81.8% of the patients had an ASA score of III.

There was no statistically significant difference in the distribution of patients’ descriptive characteristics according to the study groups (intervention-IG and control-CG) (*p* > 0.05).

### Quality of recovery-40 questionnaire

In the first measurement of the patients QoR-40; the average recovery quality level of the patients in the IG was found to be 190.06 ± 7.34; and the average recovery quality level of the patients in the CG was found to be 190.06 ± 11.59. No statistically significant difference was found between the recovery quality levels of the patients in the IG and CGs (Z= -0.942; *p* = 0.346) (Table 1). In the last measurements of the patients QoR-40 recovery quality levels; the average recovery quality level of the patients in the IG was found to be 193.57 ± 2.51 and the average recovery quality level of the patients in the CG was found to be 189.51 ± 11.06. *As a conclusion, the recovery quality level of the patients in the IG was statistically significantly higher than the patients in the CG (Z=-1.965; p  = 0.049).* In the IG compared to the first measurement was found to be statistically significant (Z=-2.550; *p* = 0.011) (Table 1). In the first face-to-face meeting, discharge education was explained to the intervention group, and a booklet and mobile application containing discharge information were given. The control group received standard hospital education.

**Table 1:** Comparison of patients’ QoR-40 survey and sub-dimension scores

QoR-40	Measurements	Statistics	Intervention (*n* = 35)	Control (*n* = 35)	Test value	*P*
Physical comfort	Baseline	Mean ± SD	55.37 ± 3.63	56.11 ± 4.23	-1.310^a^	0.190
		Median	56(48;60)	57(47;60)		
	Follow-up, 12 weeks	Mean ± SD	59.31 ± 1.57	58.14 ± 3.27	**-2.163** ^**a**^	0.031*
		Median	60(54;60)	60(48;60)		
	Difference	Mean ± SD	3.94 ± 3.77	2.03 ± 4.79	-1.896^a^	0.058
		Median	4(-1;12)	1(-11;13)		
		**Test value**	**-4.541** ^**b**^	**-2.617** ^**b**^		
		***P***	< 0.001*	0.009*		
Emotional state	Baseline	Mean ± SD	43.11 ± 2.22	41.60 ± 5.90	-0.502^a^	0.616
		Median	44(37;45)	44(15;45)		
	Follow-up, 12 weeks	Mean ± SD	44.86 ± 0.60	43.49 ± 4.19	-1.828^a^	0.068
		Median	45(42;45)	45(24;45)		
	Difference	Mean ± SD	1.74 ± 2.17	1.89 ± 7.49	-0.635^a^	0.526
		Median	1(0;8)	0(-21;30)		
		**Test value**	**-3.764** ^**b**^	**-1.995** ^**b**^		
		***P***	< 0.001*	0.046*		
Physical independence	Baseline	Mean ± SD	24.23 ± 1.66	23.83 ± 2.85	-0.106^a^	0.915
		Median	25(20;25)	25(12;25)		
	Follow-up, 12 weeks	Mean ± SD	25.00 ± 0.00	24.29 ± 2.20	**-2.540** ^**a**^	0.011*
		Median	25(25;25)	25(13;25)		
	Difference	Mean ± SD	0.77 ± 1.66	0.46 ± 3.84	-1.269^a^	0.204
		Median	0(0;5)	0(-12;13)		
		**Test value**	**-2.555** ^**b**^	-0.945^b^		
		***P***	0.011*	0.344		
Patient support	Baseline	Mean ± SD	34.34 ± 1.75	34.49 ± 1.54	-0.343^a^	0.731
		Median	35(29;35)	35(29;35)		
	Follow-up, 12 weeks	Mean ± SD	29.91 ± 0.28	29.43 ± 1.61	-0.872^a^	0.383
		Median	30(29;30)	30(23;30)		
	Fark	Mean ± SD	-4.43 ± 1.80	-5.06 ± 2.36	**-**0.780^a^	0.435
		Median	-5(-6;1)	-5(-12;1)		
		**Test value**	**-5.330** ^**b**^	**-5.332** ^**b**^		
		***P***	< 0.001*	< 0.001*		
Pain	Baseline	Mean ± SD	33.00 ± 2.40	34.03 ± 1.36	-1.893^a^	0.058
		Median	34(27;35)	35(30;35)		
	Follow-up, 12 weeks	Mean ± SD	34.49 ± 0.98	34.17 ± 1.20	-1.206^a^	0.228
		Median	35(31;35)	35(30;35)		
	Difference	Mean ± SD	1.49 ± 2.03	0.14 ± 1.67	**-2.945** ^**a**^	0.003*
		Median	1(-3;7)	0(-3;4)		
		**Test value**	**-3.662** ^**b**^	-0.359^b^		
		***P***	< 0.001*	0.719		
QoR-40 total score	Baseline	Mean ± SD	190.06 ± 7.34	190.06 ± 11.59	-0.942^a^	0.346
		Median	192(171;200)	193(148;200)		
	Follow-up, 12 weeks	Mean ± SD	193.57 ± 2.51	189.51 ± 11.06	**-1.965** ^**a**^	0.049*
		Median	195(183;195)	194(143;195)		
	Difference	Mean ± SD	3.51 ± 7.05	-0.54 ± 16.19	-1.918	0.055
		Median	3(-7;21)	-3(-54;47)		
		**Test value**	**-2.550** ^**b**^	-0.599^b^		
		***P***	0.011*	0.549		

*SD *Standard Deviation

^a^Mann-Whitney U test

^b^Wilcoxon signed-rank test

*p < 0,05

### Multidimensional quality of life scale

In the first measurement total score of MILQ-TR of the patients in the IG was 173.31 ± 34.21 points; and in the CG was 176.54 ± 32.79 points (Table 2). No statistically significant difference was found between the MILQ levels of patients in the IG and CGs (Z= -0.272; *p* = 0.786). In the last measurement total score of MILQ-TR of the patients in the IG was 204.17 ± 26.22; and in the CG was 179.54 ± 40.59 points. As a result the quality of life of patients in the IG was significantly higher than that of patients in the CG (Z=-2.857; *p* = 0.004).

**Table 2:** Comparison of patients’ Multidimensional Quality of Life Scale (MILQ-TR) and sub-dimension scores

MILQ-TR			Intervention (*n* = 35)	Control (*n* = 35)		
	Measurements	Statistics	Mean ± SD	Mean ± SD	Test value	*P*
Physical health	Baseline	Mean ± SD	66.86 ± 18.64	70,11 ± 16.24	-0.509^a^	0.611
		Median	72(28;91)	77(26;91)		
	Follow-up, 12 weeks	Mean ± SD	85.26 ± 12.98	72,20 ± 20.69	**-2.964** ^**a**^	0.003*
		Median	84(32;105)	78(26;98)		
	Difference	Mean ± SD	18.40 ± 23.40	2,09 ± 23.66	**-2.774** ^**a**^	0.006*
		Median	12(-46;74)	6(-44;50)		
		**Test value**	**-4.171** ^**b**^	-0.626^b^		
		***P***	< 0.001*	0.531		
Mental health	Baseline	Mean ± SD	19.97 ± 7.75	21.60 ± 6,23	-0.794^a^	0.427
		Median	24(4;28)	24(4;28)		
	Follow-up, 12 weeks	Mean ± SD	25.03 ± 3.80	21.80 ± 6.38	**-2.670** ^**a**^	0.008*
		Median	24(8;28)	24(4;28)		
	Difference	Mean ± SD	5.06 ± 8.69	0.20 ± 8.27	**-2.109** ^**a**^	0.035*
		Median	0(-16;21)	0(-16;20)		
		**Test value**	**-3.141** ^**b**^	-0.069^b^		
		***P***	0.002*	0.945		
Access to the health personel	Baseline	Mean ± SD	31.26 ± 2.51	31.11 ± 2.94	-0.271^a^	0.786
		Median	30(25;35)	30(22;35)		
	Follow-up, 12 weeks	Mean.±SD	31.66 ± 3,11	30.14 ± 3,50	**-2.278** ^**a**^	0.023*
		Median	30(22;35)	30(21;35)		
	Difference	Mean.±SD	0.40 ± 3,94	-0.97 ± 4,42	-1.900^a^	0.057
		Median	0(-13;7)	0(-9;13)		
		**Test value**	-1.223^b^	-1.562^b^		
		***P***	0.221	0.118		
Interpersonal relations	Baseline	Mean ± SD	27.11 ± 5.46	26.69 ± 6.58	-0.048^a^	0.962
		Median	29(12;35)	29(7;35)		
	Follow-up, 12 weeks	Mean ± SD	30.77 ± 4.64	27.03 ± 7.08	**-2.329** ^**a**^	0.020*
		Median	30(13;35)	30(8;35)		
	Difference	Mean ± SD	3.66 ± 6.47	0.34 ± 7.70	**-2.253** ^**a**^	0.024*
		Median	4(-17;23)	0(-17;23)		
		**Test value**	**-3.339** ^**b**^	-0.238^b^		
		***P***	0.001*	0.812		
Financial status	Baseline	Mean ± SD	17.71 ± 2.33	17.14 ± 2.60	-0.988^a^	0.323
		Median	18(12;21)	18(11;21)		
	Follow-up, 12 weeks	Mean ± SD	19.20 ± 1.66	17.69 ± 2.71	**-2.495** ^**a**^	0.013*
		Median	18(15;21)	18(10;21)		
	Difference	Mean ± SD	1.49 ± 2.89	0.54 ± 3.71	-1.109^a^	0.268
		Median	0(-3;9)	0(-8;9)		
		**Test value**	**-2.713** ^**b**^	-0.809^b^		
		***P***	0.007*	0.419		
Social function	Baseline	Mean ± SD	10.40 ± 2.72	9.89 ± 3.33	-0.573^a^	0.567
		Median	12(4;14)	10(2;14)		
	Follow-up, 12 weeks	Mean ± SD	12.26 ± 2.13	10.69 ± 3.14	**-2.442** ^**a**^	0.015*
		Median	12(4;14)	12(2;14)		
	Difference	Mean ± SD	1.86 ± 3.21	0.80 ± 3.62	-1.527^a^	0.127
		Median	2(-8;10)	0(-6;10)		
		**Test value**	**-3.362** ^**b**^	-0.997^b^		
		***P***	0.001*	0.319		
MILQ-TR total	Baseline	Mean ± SD	173.31 ± 34.21	176.54 ± 32.79	-0.272^a^	0.786
		Median	179(96;224)	182(88;224)		
	Follow-up. 12 weeks	Mean ± SD	204.17 ± 26.22	179.54 ± 40.59	**-2.857** ^**a**^	0.004*
		Median	198(97;238)	192(84;131)		
	Difference	Mean ± SD	30.86 ± 43.89	3.00 ± 46.98	**-2.609** ^**a**^	0.009*
		Median	25(-95;141)	6(-91;110)		
		**Test value**	**-3.796** ^**b**^	-0.486^b^		
		***P***	< 0.001*	0.627		

*SD *Standard Deviation

^a^Mann-Whitney U test

^b^Wilcoxon signed-rank test

*p < 0,05

### Discharge education knowledge level of the patients and complications

There was no statistically significant difference between the pre-test discharge education knowledge level of the patients (Z=-0.090; *p* = 0.928). In the post-test evaluation, it was seen that the discharge education knowledge level of the patients in the IG was significantly higher than that of the CG (Z=-6.105; *p* < 0.001) (Table 3).

**Table 3:** Patients’ knowledge level regarding discharge education (KLRED)

KLRED	Intervention (*n* = 35)	Control (*n* = 35)		
	Mean ± SD	Mean ± SD	Test value	*P*
Pre-test	5.71 ± 1.81	5.57 ± 2.00	Z^a^=-0.090	0.928
Post education test	9.00 ± 1.41	NA	NA	NA
Post-test	8.74 ± 1.48	5.66 ± 1.49	**Z** ^**a**^ **=-6.105**	< 0.001*
Test value/*P*	**χ2 = 45.817**/***p*** < 0.001*	NA	NA	NA
Difference 1 (Pre-test/Post-test)	3.03 ± 1.98	0.09 ± 2.11	**Z** ^**a**^ **=-5.020**	< 0.001*
	**Z**^**b**^**=-4.901/*p*** < 0.001*	Z^b^=-0.023/*p* = 0.982		
Difference 2 (Pre-test/ Post education)	3.29 ± 2.08	NA		
	**Z**^**b**^**=-4.810/*p*** < 0.001*	NA		
Difference 3 (Post education/ Post-test)	-0.26 ± 1.56	NA		
	Z^b^=-0.978/*p* = 0.328	NA		

*Ss *Standard Deviation,* NA *Not Applicable

*p < 0,05; χ2 = Friedman test, Z^a^= Mann-Whitney U test, Z^b^= Wilcoxon signed-rank test

A statistically significant difference was found between the study groups in terms of postoperative 8th-week complication rates and hospital readmission rates (*p* = 0.014 and *p* = 0.013, respectively). At the postoperative 8th week, complication and hospital readmission rates were higher in the control group compared to the experimental group.

No statistically significant difference was found between the groups regarding postoperative rehospitalization rates (*p* > 0.05) (Table 4).

**Table 4:** Comparison of patient postoperative follow-up results

	Intervention (*n* = 35)	Control (*n* = 35)	Total (*N* = 70)		
	*n*(%)	*n*(%)	*N*(%)	Test value	*P*
Complications					
Follow-up, 10 days	14(40.0)	16(45.7)	30(42.9)	0.058^a^	0.809
Follow-up, 4 weeks	12(34.3)	14(40.0)	26(37.1)	0.061^a^	0.805
Follow-up, 8 weeks	8(22.9)	19(54.3)	27(38.6)	**6.029** ^**a**^	0.014*
Follow-up, 12 weeks	8(22.9)	15(42.9)	23(32.9)	2.331^a^	0.127
Hospital admissions					
Follow-up, 10 days	6(17.1)	8(22.9)	14(20.0)	0.089^a^	0.765
Follow-up, 4 weeks	4(11.4)	7(20.0)	11(15.7)	0.431^a^	0.511
Follow-up, 8 weeks	1(2.9)	9(25.7)	10(14.3)	-^b^	0.013*
Follow-up, 12 weeks	2(5.7)	6(17.1)	8(11.4)	-^b^	0.259
Re-hospitalizations					
Follow-up, 10 days	4(11.4)	3(8.6)	7(10.0)	-^b^	0.999
Follow-up, 4 weeks	3(8.6)	2(5.7)	5(7.1)	-^b^	0.999
Follow-up, 8 weeks	0(0.0)	3(8.6)	3(4.3)	-^b^	0.239
Follow-up, 12 weeks	1(2.9)	0(0.0)	1(1.4)	-^b^	0.999

*p < 0,05; a: Yates chi-square test, b: Fisher’s Exact Test

### Structural equation modeling of the study

**Fig. 1:**
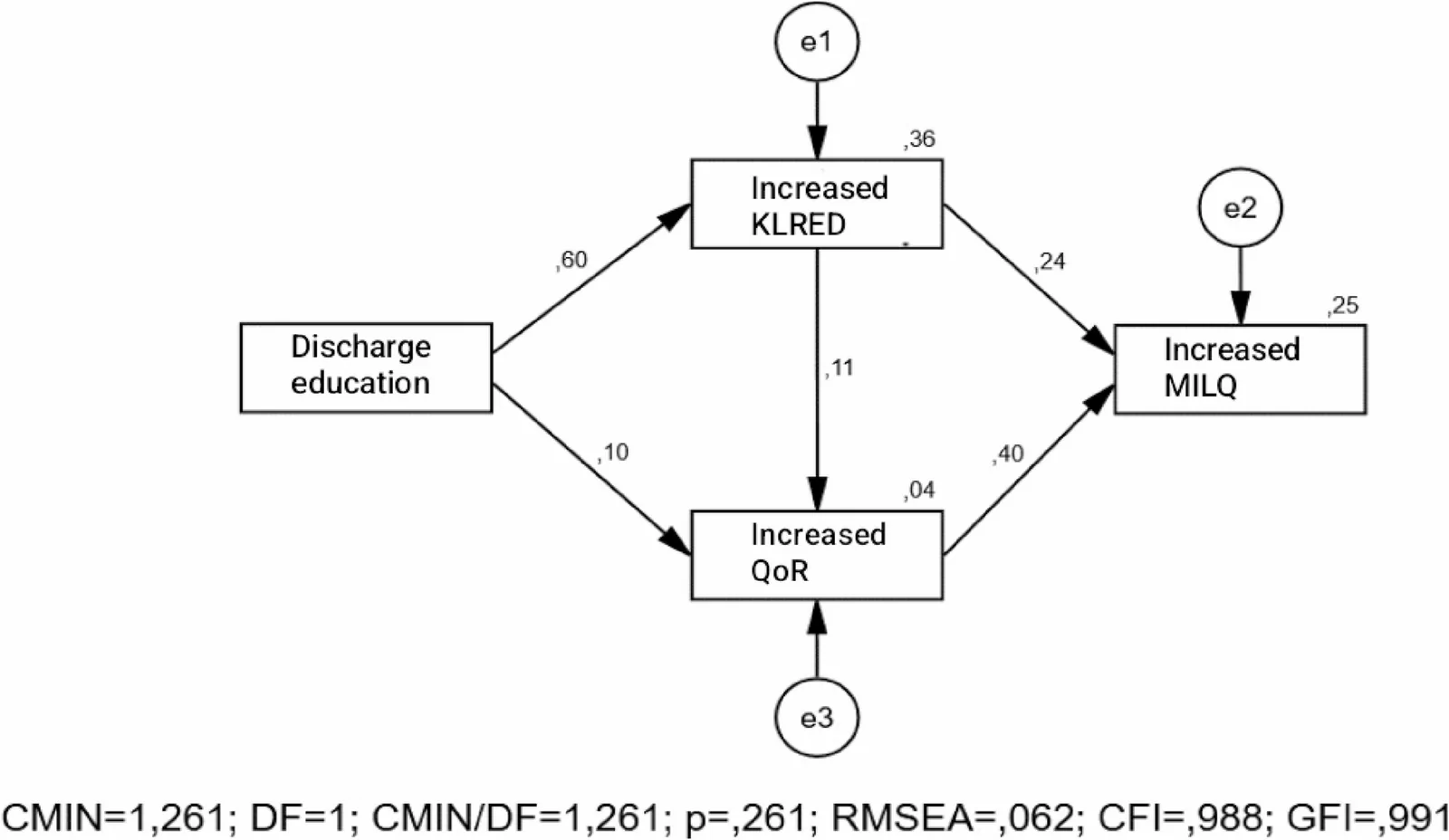
Structural Equation Modelling (SEM) of the study

Figure 1 shows the Structural Equation Modeling (SEM) diagram standardized path coefficients and error values (Fig. 1). According to SEM, discharge education increased the knowledge of the patients (β = 0.60; *p* < 0.001) and explained approximately 36% (R2 = 0.357) of the variance on the increase in knowledge of the patients. The discharge education (β = 0.10; *p* = 0.527) and increase in knowledge (β = 0.11; *p* = 0.455) did not have a statistically significant effect on the quality of recovery of the patients. The increase in knowledge (β = 0.24; *p* = 0.034) and increase in recovery quality (β = 0.40; *p* < 0.001) provided an increase in the patients’ QOL. Increased knowledge and improved recovery quality explained approximately 25% (R2 = 0.245) of the variance in patients’ QOL.

## Discussion

The aim of cardiac surgery is not only to reduce death but also to create positive improvements in recovery and QOL, this being an important goal for both patients and healthcare professionals [11, 12]. The findings and results of this study were discussed under three main headings in line with the current literature.

QoR-40 Questionnaire developed by Myles et al. (2000) by applying it to 108 adult cardiac surgery patients, showed that the scale was appropriate for use after surgery [13, 14]. In the study of Myles et al., it was found that the quality of recovery was high 3 months after cardiac surgery [14]. Similarly in this study, in the measurement applied 3 months later the recovery quality level of the patients in the IG (193.57 ± 2.51) was found to be significantly higher than that of the patients in the CG (189.51 ± 11.06) (Z=-1.965; p = 0.049). These results confirm the H1_1_ hypothesis of the study, ‘Patients who were given discharge education after cardiac surgery recover faster than those who were not given’. Thus, the positive effect of planned and model-based (ADDIE- Analysis, Design, Development, Implementation, and Evaluation) discharge education on the recovery quality of the patients was confirmed by this study’s results. Additionally, a planned discharge education and telephone follow-up will accelerate recovery and reduce complication rates, hospital admissions and re-hospitalizations of the patients.

When examining the sub-dimensions of the scale, the most significant improvements in the study by Myles et al. (2006) were seen in the sub-dimensions of physical dependency, pain, patient support and emotions [14]. In this study, improvements in the sub-dimensions of comfort (*p* = 0.031), physical dependency (*p* = 0.011) and pain (*p* = 0.003) were found to be significantly higher in the intervention group.

Most studies in the literature have reported a follow-up period limited to 3–12 months after surgery, and the results regarding recovery are similar to this study [14, 15]. It is clearly stated in the literature that most cardiac surgery patients achieve a good recovery and QOL as of the 3rd month after surgery and maintain their well-being for at least three years [14, 16]. Since this study had a 12-week follow-up period, longer term results could not be examined.

In Yolcu and Akin’s (2015) study, where the research sample consisted of 80 patients who underwent orthopaedic, cardiac and general surgeries, the QoR-40 survey scores of orthopaedic and general surgery patients were found to be significantly higher than the scores of patients who underwent cardiac surgery [15]. According to Yolcu and Akın’s study, patients who underwent cardiac surgery needed support in issues such as pain control, mobilisation, emotional state and physical comfort. In this study, significant improvements were achieved in the sub-dimensions of physical comfort, mobilisation and pain in the intervention group, and in this context, it can be said that discharge education provided improvement in the most needed issues.

### Multidimensional quality of life scale

In the literature, studies evaluating QOL after cardiac surgery are insufficient, and no studies investigating the effect of discharge education on QOL were found. In addition, the MILQ scale was newly translated into Turkish by Demir and Özer (2019) and only the authors study with 370 patients with acute coronary syndrome is available. According to this study, it was concluded that the Turkish version of the MILQ scale is an effective tool in measuring QOL despite cultural differences [17].

The MILQ scale is developed specifically for patients with CVD. In the literature, it is mostly used on cardiology patients [17–21]. However, in the scale development study, 43.0% of the sample consisted of patients who had undergone CABG surgery, and the German translation was applied to patients who had undergone CABG surgery [22, 23].

Mastrigt et al. (2010) applied the MILQ scale to measure the QOL of 410 patients who underwent CABG surgery and applied a short-term intensive care unit stay package (> 8 h). In the measurements they made in the first month after the surgery, they found a significant difference in the mean scores of the physical health and social function sub-dimensions of the experimental group. However, in the second measurement made 1 year later, no difference was found between the groups [23]. In our study, the measurement made in the 3rd month, showed a significant difference between the groups in the total (*p* = 0.009) and sub-dimensions (*p* = 0.006; *p* = 0.035; *p* = 0.024) scores. The lack of significant results in the Mastrigt et al. (2019) study may be associated with the lack of a comprehensive discharge education plan as in our study and the presence of a single variable (> 8 h in CVICU).

In the study conducted by Strada et al. (2013) on patients with advanced heart and lung disease, the first measurement MILQ composite score was found to be 53 and 34% of the patients were seen to have a moderate-low score (MILQ < 48) [20]. Similarly, in the study conducted by Blinderman et al. (2008) on patients with advanced congestive heart disease, the median MILQ composite score was found to be 56 [21]. In this study, the mean MILQ-TR in the IG at the first measurement was 173.31 ± 34.21; the mean MILQ-TR in the CG was 176.54 ± 32.79; in the last measurement, the mean MILQ-TR in the IG was 204.17 ± 26.22; the mean MILQ-TR in the CG was 179.54 ± 40.59, which is moderate-high level. Thus, the positive effect of the discharge education was proven by the fact that the final measurement results in the IG were significantly higher (Z=-2.857; *p* = 0.004). Based on these results, it can be said that cardiac surgery affects permanently and positively both, the physical and mental components of the QOL.

The final evaluation of MILQ-TR shows that the average QOL level of the patients in the IG (204.17 ± 26.22) was significantly higher than that of the patients in the CG (179.54 ± 40.59) (Z= -2.857; *p* = 0.004). The difference of scores between the two groups was found significantly greater in the IG (Z= -2.609; *p* = 0.009). All sub-dimensions results are also parallel to the results obtained from the general scores of the MILQ-TR scale. These results confirm the 3rd hypothesis H1_3_ of this study: ‘The QOL of patients who were given discharge education after cardiac surgery is higher than those who were not given.’ The importance of planned and continuous education is supported by the results of this study.

### Discharge education knowledge level of the patients

Despite all efforts, the educational needs of surgical patients have not yet been met [24–26]. According to the results of a meta-analysis, individual education given to cardiac surgery patients improves the patient’s QOL, provides positive changes in health behaviours, and significantly reduces anxiety and depression [27]. Işık and Karaöz (2022), found that discharge education and counselling services positively affect individuals’ care habits, reduce problems experienced after discharge, and increase the level of self-efficacy [28]. Another study emphasizes that, after cardiac surgery, home based education and a new lifestyle is necessary to reduce patients’ anxiety levels and should be implemented as a part of surgical and medical treatment [29]. In this study’s post-test results, the discharge education knowledge level of the patients in the IG was significantly higher than that of the CG (Z=-6.105; *p* < 0.001). The difference between the pre-test and post-test measurements shows that the discharge knowledge level of the patients in the IG increased by an average of 3.03 ± 1.98 units, while that of the patients in the CG increased by an average of 0.09 ± 2.11 units. The difference between the two groups scores shows that the increase in the IG was significantly higher than the CG (Z=-5.020; *p* < 0.001). The study results were similar to the literature. According to these results, the discharge education supported by the education booklet and mobile application was found effective in increasing the knowledge level of the patients. In this study, all the patients in the IG (100%) stated that they benefited from discharge education. The level of patients benefiting from discharge education was calculated as an average of 8.57 ± 1.87 points out of 10. In this study, 11.4% of the IG patients stated that they benefited from the mobile application. According to these findings, the benefits of discharge education are reflected in both patient statements and knowledge test levels.

According to the literature, postoperative CABG patients need information on topics such as: pain management, medication use, body hygiene, nutrition, excretion, wound care, maintaining effective breathing, starting work, daily living activities and exercises, smoking cessation, alcohol, sports, routine check-ups, driving and sexuality [23]. The most frequently repeated topics in this study are similar to the literature: respiratory exercise, pain management, use of anti-embolic socks, rest, nutrition/water, termination of sternum precautions, exercise/walking, medication management, bathing-personal hygiene, sternum wound healing.

Discharge education given in the hospitals before discharge is not sufficient to meet the patient’s needs at home so they need further support like tele nursing. In the study of Tuna and Emre (2020), it was found that nurse counselling by phone was positively agreed by the patients and had a positive effect on care outcomes. According to the data of the Home Tele-Nursing Program, which Guzman-Clark et al. (2021) started to use in the early 2000s, there was a 59% decrease in the length of hospital stay and a 31% decrease in hospital admissions in 2016 for registered heart failure veterans [30]. Albayram et al. (2022) concluded that continuity of telephone counselling and discharge education after heart surgery is important, positively affects patients and improves care after heart surgery [31]. In our study, patients were called by the researcher 4 times, the average length of call on the 10th day after surgery was 187.03 ± 137.82 s, 192.64 ± 146.64 s on the 4th week, 256.73 ± 224.08 s on the 8th week, and 531.39 ± 253.35 s on the 12th week after surgery. The effectiveness of the phone calls in this study could not be measured with a special scale, but the fact that 100% of the patients stated that they benefited from the education and gave a score of 8.57 ± 1.87 out of 10 is similar to other studies in this context.

As a result, regardless of the communication tools, close support during the home recovery process after discharge, open and quality communication with healthcare professionals such as physicians and nurses are effective in coping with the disease process of CV patients and in general all of surgery patients [32].

## Conclusion and recommendations

The aim of the study was to examine the effects of discharge education given after cardiac surgery on quality of life and recovery. As a result of this study, patients who were given discharge education after cardiac surgery recovered faster than those who were not given, they had a higher QOL and fewer complication rates. Additionally, discharge education supported by booklet and mobile application increased the knowledge level of patients, telenursing was an effective method in terms of follow up, counselling and repetition of education, the increase in patients’ knowledge provided a significant increase in QOL (MILQ-TR) and the increase in the quality of recovery (QoR-40) of patients provided a significant increase in quality of life (MILQ-TR).

In line with the results stated above, it is recommended to nurses to: include innovative and digital tools in addition to traditional methods such as booklets and written materials in patient discharge education, evaluate the QOL level of patients who underwent cardiac surgery, provide education and training pre-, peri-, postoperatively and at discharge in major surgeries such as cardiac surgery, prepare personalized education programs for risk groups, such as patients with chronic diseases and morbidities, in order to prevent complications and increase QOL, closely monitor patients who have undergone cardiac surgery for at least 12 weeks after discharge, repeat discharge education when needed, and integrate telenursing and current technology into the existing health system.

## Supplementary Information

Supplementary Material 1.

Supplementary Material 2.

## Data Availability

The datasets generated and/or analysed during the current study are not publicly available due to language barrier as the study is done in Turkish and due to privacy reasons, but are available from the corresponding author on reasonable request.

## Abbreviations

CVD: Cardiovascular Diseases
CAD: Cardiac Artery Diseases
CABG: Cardiac Artery Bypass Grafting
ASA: The American Society of Anaesthesiologists
CVICU: Cardiovascular Intensive Care Unit
DTKLF: Discharge Education Knowledge Level Form
QoL: Quality of Life
MILQ: Multidimensional Quality of Life Scale
QoR: 40-Quality of Recovery-40 Questionnaire
ADDIE: Analysis, Design, Development, Implementation, and Evaluation
