# Supplementary material for: Effects of discharge education program on recovery and quality of life after cardiac surgery: a 12 week follow-up single-blind randomized controlled trial
Source: BMC Surg. 2026 Jun 16;26:572. doi: 10.1186/s12893-026-03939-4 (PMC13508258; doi:10.1186/s12893-026-03939-4)
Supplement: Supplementary file 1 [file 12893_2026_3939_MOESM1_ESM.docx]

**Sudy sample (N=77)**

Inclusion criteria**:**

- Willing to participate in the study,
- Having undergone open heart surgery,
- Being 18 years or older,
- Ability to read, write, understand and communicate in Turkish,
- No vision, hearing and perception problems,
- No neurological disorder.

Population of the study

Patients hospitalized in Cardiovascular Surgery Units who met the inclusion criteria

Control Group (n=38)

On the 5th-7th day after surgery: Patient’s Personal Information Form, Multidimensional Quality of Life Form, Recovery Quality-40 Form, Pre- test

Week 12 phone interview

Multidimensional Quality of Life Form, Quality of Recovery- 40 Form, Post-test

10th day phone call (review of education topics if asked)

4th week phone call (review of education topics if asked)

8th week phone call (review of education topics if asked)

Control Group (n=35)

Intervention Group(n=39)

Post-education test

10th day phone call (review of required education topics)

4th week phone call (review of required education topics)

8th week phone call (review of required education topics)

Week 12 phone interview

Multidimensional Quality of Life Form, Quality of Recovery- 40 Form, Post-test

Permuted Block Randomization

On the 5th-7th day after surgery: Patient’s Personal Information Form, Multidimensional Quality of Life Form, Recovery Quality-40 Form, Pre-education test

No intervention-Routine hospital discharge education

Discharge Training prepared by the researcher

Intervention Group(n=35)

Flowchart 1. Flowchart of the Study
