# Supplementary material for: Effects of discharge education program on recovery and quality of life after cardiac surgery: a 12 week follow-up single-blind randomized controlled trial
Source: BMC Surg. 2026 Jun 16;26:572. doi: 10.1186/s12893-026-03939-4 (PMC13508258; doi:10.1186/s12893-026-03939-4)
Supplement: Supplementary file 2 [file 12893_2026_3939_MOESM2_ESM.docx]

Table 1. Comparison of Demographic and Clinical Characteristics of the Groups

| **Variables** | **Category** | **Intervention (n=35) n(%)** | **Control (n=35) n(%)** | **Total (n=70) n(%)** | **Test value** | **p-value** |
| --- | --- | --- | --- | --- | --- | --- |
| Age group | ≤60 | 21 (60.0) | 12 (34.3) | 33 (47.1) | 3.669c | 0.055 |
|  | >60 | 14 (40.0) | 23 (65.7) | 37 (52.9) |  |  |
| Gender | Male | 28 (80.0) | 26 (74.3) | 54 (77.1) | 0.081c | 0.776 |
|  | Female | 7 (20.0) | 9 (25.7) | 16 (22.9) |  |  |
| BMI | Mean±SD | 27.24±4.10 | 27.80±3.72 | 27.52±3.90 | 0.604a | 0.548 |
| Education level | Below high school | 26 (74.3) | 23 (65.7) | 49 (70.0) | 0.272c | 0.602 |
|  | High school and above | 9 (25.7) | 12 (34.3) | 21 (30.0) |  |  |
| Marital status | Married | 31 (88.6) | 31 (88.6) | 62 (88.6) | -d | 1.000 |
|  | Single | 4 (11.4) | 4 (11.4) | 8 (11.4) |  |  |
| Place of residence | Village/town | 1 (2.9) | 3 (8.6) | 4 (5.7) | 1.594d | 0.527 |
|  | District | 30 (85.7) | 30 (85.7) | 60 (85.7) |  |  |
|  | City | 4 (11.4) | 2 (5.7) | 6 (8.6) |  |  |
| Employment status | Employed | 17 (48.6) | 9 (25.7) | 26 (37.1) | 5.128b | 0.077 |
|  | Unemployed | 8 (22.9) | 16 (45.7) | 24 (34.3) |  |  |
|  | Retired | 10 (28.6) | 10 (28.6) | 20 (28.6) |  |  |
| Income | Moderate | 34 (97.1) | 33 (94.3) | 67 (95.7) | -d | 0.999 |
|  | Low | 1 (2.9) | 2 (5.7) | 3 (4.3) |  |  |
| Living arrangement | Alone | 3 (8.6) | 4 (11.4) | 7 (10.0) | 2.512d | 0.493 |
|  | With spouse | 10 (28.6) | 13 (37.1) | 23 (32.9) |  |  |
|  | With spouse and children | 21 (60.0) | 15 (42.9) | 36 (51.4) |  |  |
|  | With children | 1 (2.9) | 3 (8.6) | 4 (5.7) |  |  |
| Family history of open-heart surgery | No | 9 (25.7) | 14 (40.0) | 23 (32.9) | 1.619c | 0.309 |
|  | Yes | 26 (74.3) | 21 (60.0) | 47 (67.1) |  |  |
| Caregiver education (hospital) | Below high school | 21 (60.0) | 17 (48.6) | 38 (54.3) | 0.518c | 0.472 |
|  | High school and above | 14 (40.0) | 18 (51.4) | 32 (45.7) |  |  |
| Caregiver education (post-discharge) | Below high school | 22 (62.9) | 18 (51.4) | 40 (57.1) | 0.525c | 0.469 |
|  | High school and above | 13 (37.1) | 17 (48.6) | 30 (42.9) |  |  |
| Chronic disease | No | 5 (14.3) | 7 (20.0) | 12 (17.1) | 0.101c | 0.751 |
|  | Yes | 30 (85.7) | 28 (80.0) | 58 (82.9) |  |  |
| Chronic disease type | Hypertension (HT) | 16 (45.7) | 20 (57.1) | 36 (51.4) | 0.515c | 0.473 |
|  | Diabetes Mellitus (DM) | 15 (42.9) | 14 (40.0) | 29 (41.4) | 0.000c | 0.999 |
|  | Cholesterol | 4 (11.4) | 5 (14.3) | 9 (12.9) | -d | 0.999 |
|  | Coronary Artery Disease (CAD) | 10 (28.6) | 6 (17.1) | 16 (22.9) | 0.729c | 0.393 |
| Previous hospitalization | No | 7 (20.0) | 9 (25.7) | 16 (22.9) | 0.081c | 0.776 |
|  | Yes | 28 (80.0) | 26 (74.3) | 54 (77.1) |  |  |
| Regular medication use | No | 8 (22.9) | 5 (14.3) | 13 (18.6) | 0.378c | 0.539 |
|  | Yes | 27 (77.1) | 30 (85.7) | 57 (81.4) |  |  |
| Non-prescription drug use | No | 34 (97.1) | 33 (94.3) | 67 (95.7) | -d | 0.999 |
|  | Yes | 1 (2.9) | 2 (5.7) | 3 (4.3) |  |  |
| Smoking | No | 11 (31.4) | 10 (28.6) | 21 (30.0) | 0.000c | 0.999 |
|  | Yes | 24 (68.6) | 25 (71.4) | 49 (70.0) |  |  |
| Alcohol use | No | 30 (85.7) | 27 (77.1) | 57 (81.4) | 0.378c | 0.539 |
|  | Yes | 5 (14.3) | 8 (22.9) | 13 (18.6) |  |  |
| ASA | I | 1 (3.1) | 1 (2.9) | 2 (3.0) | 0.600d | 0.865 |
|  | II | 4 (12.5) | 6 (17.6) | 10 (15.2) |  |  |
|  | III | 27 (84.4) | 27 (79.4) | 54 (81.8) |  |  |
| Surgical procedure | CABG | 29 (82.9) | 32 (91.4) | 61 (87.1) | -d | 0.477 |
|  | Other | 6 (17.1) | 3 (8.6) | 9 (12.9) |  |  |
